# ^18^FDG-PET/CT for predicting the outcome in ER+/HER2- breast cancer patients: comparison of clinicopathological parameters and PET image-derived indices including tumor texture analysis

**DOI:** 10.1186/s13058-016-0793-2

**Published:** 2017-01-05

**Authors:** David Groheux, Antoine Martineau, Luis Teixeira, Marc Espié, Patricia de Cremoux, Philippe Bertheau, Pascal Merlet, Charles Lemarignier

**Affiliations:** 1 Department of Nuclear Medicine, Saint-Louis Hospital, Paris, France; 2 University Paris-Diderot, INSERM/CNRS UMR944/7212, Paris, France; 3 Breast Diseases Unit, Saint-Louis Hospital, Paris, France; 4 Department of Biochemistry, Saint-Louis Hospital, Paris, France; 5 Department of Pathology, Saint-Louis Hospital, Paris, France

**Keywords:** ER+/HER2- breast cancer, Prognosis, ^18^FDG-PET/CT, SUV, Metabolically active tumor volume, Textural parameters

## Abstract

**Background:**

This study investigated the value of some clinicopathological parameters and 18 F-fluorodeoxyglucose-positron emission tomography/computed tomography (^18^FDG-PET/CT) indices, including textural features, to predict event-free survival (EFS) in estrogen receptor-positive/human epidermal growth factor receptor 2-negative (ER+/HER2-) locally advanced breast cancer (BC) patients.

**Methods:**

FDG-PET/CT indices and clinicopathological parameters were assessed before neoadjuvant chemotherapy (NAC). After completion of chemotherapy, all patients had breast surgery with axillary lymph node dissection, followed by radiation therapy and endocrine therapy for 5 years. EFS was estimated using the Kaplan-Meier method. A Cox proportional hazard regression model was used for multivariate analysis.

**Results:**

One hundred forty-three consecutive patients with stage II–III ER+/HER2- BC and without distant metastases at baseline PET were included. High standardized uptake values (SUVs), were associated with shorter EFS (HR = 3.51, *P <* 0.01 for SUV_max_; HR = 2.76*, P =* 0.02 for SUV_mean_; and HR = 4.40 *P* < 0.01 for SUV_peak_). Metabolically active tumor volume (MATV, HR = 3.47, *P <* 0.01) and total lesion glycolysis (TLG, HR = 3.10, *P <* 0.01) were also predictive of EFS. Homogeneity was not predictive (HR = 2.27, *P =* 0.07) and entropy had weak prediction (HR = 2.89, *P =* 0.02). Among clinicopathological parameters, EFS was shorter in progesterone receptor (PR)-negative tumor (vs*.* PR-positive tumor; HR = 3.25, *P <* 0.01); histology was predictive of EFS (lobular vs. ductal invasive carcinoma; HR = 3.74, *P =* 0.01) but not tumor grade (grade 3 vs*.* grade 1–2; HR = 1.64, *P* = 0.32). Pathological complete response after NAC was not correlated to the risk of relapse. Three parameters remained significantly associated with EFS in multivariate analysis. MATV (HR = 1.01, *P* < 0.01), progesterone receptor expression (HR = 2.90, *P =* 0.03) and tumor histology (HR = 3.80, *P =* 0.02).

**Conclusions:**

Baseline PET parameters measured before neoadjuvant treatment have prognostic values in ER+/HER2- locally advanced breast cancer patients. After multivariate analysis, metabolically active tumor volume remains significant while textural analysis of PET images is not of added value. Considering histopathological parameters, our study shows that patients with PR-negative or lobular invasive tumor have poorer prognosis than patients with PR-positive or ductal carcinoma, respectively.

## Background

Neoadjuvant chemotherapy (NAC) is commonly used for patients with large or locally advanced breast cancer (BC). Pathological complete response (pCR) after NAC (absence of residual invasive cells in the primary tumor and axillary lymph nodes) is correlated with better survival [1]. This relationship between pathological response and outcome is stronger in aggressive subtypes such as the triple-negative (TN) BCs than in estrogen receptor-positive/human epidermal growth factor receptor 2-negative (ER+/HER2-) BCs [1]. Moreover, by definition, pCR can be only determined at the end of the NAC. Various clinical and biological BC characteristics assessed before treatment have shown a prognostic value. For example, patients with lymph node involvement have poorer prognosis than patients without lymph node involvement (N0) and higher-grade tumors are more aggressive than lower-grade tumors.

Positron emission tomography/computed tomography (PET/CT) imaging could provide additional prognostic information. It has been suggested that high baseline ^18^F-fluorodeoxyglucose (^18^FDG) uptake assessed by high standardized uptake value (SUV) could be associated with poor prognostic factors such as the high histological grade [2] as well as worse survival [3, 4]. These previous studies were focused on the prognostic value of SUV_max_, without testing other PET parameters. However, the prognostic value of SUV measurement could be more limited in the ER+/HER2- subtype than in other subtypes because luminal tumors are somewhat less ^18^FDG-avid [2]. PET parameters, taking into account metabolic volume measurements, such as total lesion glycolysis (TLG), may improve risk stratification in this specific subtype [5–8].

Recently, tumor textural analysis and heterogeneity assessed with PET emerged also as potential prognostic factors in BC [9, 10]. These studies [9, 10] included patients with different subtypes of breast cancer (triple-negative tumors mixed with HER2+ tumors and/or with ER+ tumors), receiving mixed treatments. Only one study examined the relation between textural features and patient outcome [10].

Our study was focused in a homogenous and large group of 143 patients with ER+/HER2- breast cancer, all receiving the same treatment. We tested not only the prognostic value of SUV_max_ but also the value of SUV_mean_, SUV_peak_, metabolically active tumor volume (MATV), total lesion glycolysis, and novel parameters characterizing the tumor texture [homogeneity (H) and entropy (E)]. The prognostic value of these PET features was compared to clinicopathological prognostic factors.

## Methods

### Study design

We performed ancillary analysis of prospectively acquired data in the frame of the ASAINT study (NCT02599974). ASAINT aimed at evaluating the diagnostic performance and the prognostic value of ^18^FDG-PET/CT in patients with stage II or III breast cancer undergoing neoadjuvant chemotherapy. The main objective of the present study was to compare the value of some clinicopathological parameters and PET-derived image indices in predicting event-free survival (EFS) in the subgroup of ER+/HER2- breast carcinoma. The secondary objective was to examine the association between baseline PET image parameters and tumor characteristics.

The study was approved by the institutional review board with waivers of informed written consent for this ad hoc analysis of image-derived data.

### Tumor histology and immunohistochemistry

Breast cancer diagnosis was performed on a core needle biopsy before neoadjuvant chemotherapy. Tumor grade used the modified Scarff-Bloom-Richardson (SBR) system. Tumors were estrogen receptor positive (ER+) if showing moderate or high positivity (2+ or 3+) of at least 10% of cells. The same criteria were used for progesterone receptor (PR). Tumors were HER2+ if more than 30% of cells showed definite membrane staining [11]. Control by fluorescence in situ hybridization or silver in situ hybridization was done for ambiguous cases. Only patients with ER+/HER2- tumors were included.

### ^18^FDG-PET/CT imaging

Patients fasted for 6 hours and blood glucose level had to be less than 7 mmol/L. Imaging started 60 minutes after injection of ^18^FDG (5 MBq/kg); a 2 minutes per bed acquisition was performed from mid-thigh level to the base of the skull with the arms raised. The same PET/CT scanner was used for all the patients: the Gemini XL (Philips Medical Systems, Amsterdam, The Netherlands). CT data were acquired first (120 kV; 100 mAs; 0.813 pitch factor; no contrast enhancement). CT images were reconstructed with a pixel size of 1.17 mm. Reconstruction section thickness was 3 mm and the reconstruction interval was 2 mm. PET images were reconstructed with a isotropic voxel size of 4 mm. Accurate registration of CT reconstructed and PET reconstructed images was obtained by annual system alignment calibration of the bed, CT gantry, and PET gantry. The attenuation-corrected PET images were normalized for injected dose and body weight, and subsequently converted into standardized uptake values (SUVs), defined as: [tracer concentration (kBq/mL)] / [injected activity (kBq)/patient body weight (g)].

### PET-derived metabolic indices

For each patient, a large region of interest (ROI) encompassing the primary tumor and healthy breast tissue surrounding the tumor was delineated manually by a nuclear medicine specialist. The CT information was not used for this delineation, and ROIs were drawn only in PET images. Then, the metabolically active tumor volume (MATV) was automatically extracted from the manual delineation using software based on an adaptive threshold method [12]. SUV_max_ (value of the voxel with the highest SUV), SUV_peak_ (the mean of voxel intensities in the region with the highest uptake corresponding to a spherical ROI of 1 cm^3^ [13])_,_ and SUV_mean_ (mean value of all the voxels within the segmented tumor) were automatically calculated from the adaptive threshold segmentation. Total lesion glycolysis (TLG) was defined as MATV × SUV_mean_.

The texture analysis was performed on PET images without using CT information. Texture parameters were defined for each patient on the MATV. First, SUV values were rescaled in 64 discrete bins [14]. We chose 64 bins for the rescaling of the intensity values because a previous study showed this value was optimal for textural analysis of PET images [14]. Then, three-dimensional co-occurrence matrices were calculated from resampled intensity volumes for a distance of one voxel (i.e., for pairs of adjacent voxels).

Two textural parameters, entropy and homogeneity, based on their robustness, their reproducibility, and their potentiality to characterize tumor tissues and predict patient outcome, according to previous studies [9, 15, 16] were calculated from three-dimensional co-occurrence matrices and analyzed.

Entropy (E) quantifies the randomness of voxel intensity distribution within the volume. The value of E is expected to be high if the intensity of different voxels is distributed randomly.

$$ E=-{\displaystyle \sum_{i=1}^{64}{\displaystyle \sum_{j=1}^{64}P\left[i,\ j\right] \ln P\left[i,\ j\right]}} $$

*P[i,j]* is the number of voxel pairs having intensity *i* and *j* in the co-occurrence matrix.

Homogeneity (H) quantifies the local homogeneity of a pair of voxels. The value of H is high if the intensities of each pair of voxels are similar.

$$ H={\displaystyle \sum_{i=1}^{64}{\displaystyle \sum_{j=1}^{64}\frac{P\left[i,\ j\right]}{1+\left|i-j\right|}}} $$

### Treatment

Patients received neoadjuvant chemotherapy with EC-D (four cycles of epirubicin 75 mg/m^2^ plus cyclophosphamide 750 mg/m^2^ administered every 3 weeks, followed by four courses of docetaxel 100 mg/m^2^). No neoadjuvant endocrine therapy was used. After completion of NAC, all the patients underwent breast surgery (breast-conserving surgery or mastectomy according to NAC response) with axillary lymph node dissection. After surgery, patients received locoregional radiation therapy (tailored to disease stage and breast surgery results) and adjuvant endocrine therapy for 5 years (tamoxifen in premenopausal women or aromatase inhibitors in postmenopausal women).

### Pathology assessment and patient outcome

Pathological complete response (pCR) was defined as no evidence of residual invasive cancer in breast tissues and lymph nodes (ypT0/is ypN0). Absence of carcinoma in situ was not mandatory to define pCR [1].

Date of baseline PET acquisition was considered as the beginning of follow-up.

Event-free survival (EFS) was examined in patients free of distant metastases at baseline staging. Events included local, regional, or distant recurrences or death, whichever occurred first. During neoadjuvant chemotherapy, patients were examined each two cycles. After breast surgery, patients had follow-up clinical visits every 4 months for 2 years, then twice yearly.

### Statistical analysis

All distributions were expressed as median for quantitative data or count (percentage) for categorical data. Associations between baseline tumor PET-derived images parameters (SUV_max_, SUV_mean_, SUV_peak_, MATV, TLG, homogeneity, and entropy) values and clinical or pathological/biological parameters (tumor grade, progesterone receptor expression, etc.) were examined with the Wilcoxon rank sum test. Multiple corrections were performed with the method of Benjamini-Hochberg.

Survival curves were estimated using the Kaplan-Meier method. The relation between some clinicopathological tumor characteristics and EFS were examined with the log-rank test. We also used the log-rank test to examine the relation between PET-derived image parameters and EFS. Optimal PET parameter cutoff values for predicting EFS were determined at 3 years of follow-up with the Youden index after receiver-operating characteristics curve analysis. Log-rank tests have been controlled using the Hochberg’s procedure and hazard ratios (HRs) were calculated with their 95% confidence interval (CI).

We also performed a univariate analysis using Cox proportional hazards logistic regression to identify prognostic factors for EFS. Age and PET parameters were entered as continuous variables. Then, on the basis of the univariate analysis, we performed a stepwise multivariate Cox analysis to identify independent prognostic factors. We used a stepwise forward selection approach in which the variable with the strongest association with EFS was entered first, followed sequentially by other significant variables. The variables were removed if they became nonsignificant.

All tests were two-sided and *P* values below or equal to 0.05 were considered statistically significant. Analyses were performed using R statistical software (version 3.2.2) (R Foundation for Statistical Computing, Vienna, Austria).

## Results

From June 2006 to November 2015, 146 consecutive patients with clinical stage II or III ER+/HER2- BC prospectively underwent ^18^FDG-PET/CT scanning before starting NAC. Three patients were excluded because of slight or no ^18^FDG uptake. The tumors of these three patients could not be delineated. Table 1 shows the main characteristics of the 143 included patients. The median follow-up period was 44 months.

**Table 1:** Overall characteristics of 143 ER+/HER2- breast cancer patients without distant metastases

	No. of patients (%)
Age in years	
mean (SD), median [min, max]	51.4 (11.4), 50 [30; 78]
Histological type	
Invasive ductal carcinoma	129 (90%)
Lobular	7 (5%)
Other	7 (5%)
Grade	
Grade 1	7 (5%)
Grade 2	91 (64%)
Grade 3	42 (29%)
Grade unknown	3 (2%)
Progesterone receptor expression	
Negative	55 (38%)
Positive	88 (62%)
Tumor classification^a^	
T1	3 (2%)
T2	53 (37%)
T3	56 (39%)
T4	31 (22%)
Lymph node classification^a^	
N0	58 (40%)
N1	71 (50%)
N2	11 (8%)
N3	3 (2%)
Stage^a^	
Stage IIA	25 (18%)
Stage IIB	49 (34%)
Stage IIIA	36 (25%)
Stage IIIB	30 (21%)
Stage IIIC	3 (2%)

*ER+/HER2-* estrogen receptor-positive/human epidermal growth factor receptor 2-negative, *SD* standard deviation

^a^Clinical classification and stage after the clinical examination and before ^18^F-FDG-PET/CT according to the seventh edition of the AJCC Cancer Staging Manual

### Relation between baseline tumor characteristics and PET-derived indices

Patient age was not associated with SUV parameters, MATV, and textural features, for the different subgroups tested (≤40y vs. >40y and ≤50y vs*.* >50y) (Table 2).

**Table 2:** Relation between some clinical or tumor characteristics and tumor PET-derived image parameters in 143 patients with ER+/HER2- breast cancer

		Age				Tumor^a^		Lymph nodes^a^		Histology		Grade		PR expression	
		≤40	>40	≤50	>50	T2	T3	N0	N+	IDC	ILC	1–2	3	Neg	Pos
No. patients		27	116	74	69	53	56	58	85	129	7	98	42	55	88
SUV_max_	med	5.4	6.8	6.5	6.6	5.2	5.9	6.1	6.6	6.8	4.8	5.4	7.9	7.2	5.7
	*P*	0.31		0.63		0.56		0.79		0.43		**<0.01**		0.06	
SUV_mean_	med	3.1	3.6	3.4	3.6	2.9	3.3	3.5	3.5	3.6	3.0	3.1	4.2	4.0	3.2
	*P*	0.36		0.67		0.59		0.77		0.31		**<0.01**		0.06	
SUV_peak_	med	4.3	5.3	5.1	5.1	4.1	4.9	4.9	5.1	5.2	4.4	4.4	6.4	5.7	4.5
	*P*	0.32		0.60		0.44		0.81		0.63		**<0.01**		0.05^b^	
MATV	med	10.1	11.2	11.0	9.6	6.7	10.8	9.2	11.3	10.1	32.5	10.9	10.4	13.9	9.1
	*P*	0.33		0.86		**<0.01**		0.24		0.02^b^		0.83		0.03^b^	
TLG	med	27.2	39.0	33.5	35.2	20.1	33.5	32.7	40.5	32.8	74.6	32.1	44.8	46.3	28.2
	*P*	0.22		0.86		**<0.01**		0.41		0.16		0.05^b^		0.01^b^	
H	med	0.12	0.12	0.12	0.12	0.11	0.12	0.12	0.12	0.12	0.14	0.12	0.12	0.13	0.12
	*P*	0.81		0.87		**0.02**		0.10		0.09		0.73		0.12	
E	med	3.5	3.8	3.7	3.6	3.2	3.7	3.5	3.8	3.6	4.8	3.7	3.7	4.0	3.5
	*P*	0.18		0.97		**<0.01**		0.25		0.02^b^		0.70		0.02^b^	

*P* value for comparison of median values with the Wilcoxon rank sum test. Bold numerals correspond to *P* values keeping statistically significant after multiple corrections from Benjamini-Hochberg

*PET* positron emission tomography, *ER+/HER2-* estrogen receptor-positive/human epidermal growth factor receptor 2-negative, *PR* progesterone receptor, *IDC* invasive ductal carcinoma, *ILC* invasive lobular carcinoma, *SUV* standardized uptake value, *MATV* metabolically active tumor volume, *TLG* total lesion glycolysis, *H* homogeneity, *E* entropy

^a^Clinical classification according to the seventh edition of the AJCC Cancer Staging Manual

^b^Not significant after correction for multiple testing

Grade 3 tumors showed higher uptake than lower grade (grade 1 + grade 2) tumors (median SUV_max_ 7.9 vs. 5.4, median SUV_mean_ 4.2 vs*.* 3.1, median SUV_peak_ 6.4 vs*.* 4.4, *P* < 0.01 in all the cases). ^18^FDG uptake seemed to be higher in invasive ductal carcinoma (IDC) than in invasive lobular carcinoma (ILC) but the difference was not significant (Table 2); only seven patients had ILC. Neither tumor characteristics according to the TNM classification of malignant tumors (clinical T score) nor lymph node characteristics according to the TNM classification (N score) were associated with the degree of ^18^FDG tumor uptake (Table 2).

On the contrary, as expected, MATV and TLG were significantly higher in T3 (tumor size > 5 cm) than in T2 tumor (tumor size ≤ 5 cm). Similarly, entropy and homogeneity were associated with tumor size. After correction for multiple testing, no significant relation was observed between the textural features and the biological or histological tumor characteristics (Table 2).

### Relation between baseline tumor characteristics or pathology findings after NAC and EFS

Twenty patients relapsed. Distant metastases were detected in 15 patients. Sites of distant metastases were bone (N = 9), liver (N = 7), distant lymph nodes (N = 4), pleura (N = 2), brain (N = 2), and skin (N = 1). Four patients with distant metastases died.

T score based on clinical examination before ^18^FDG-PET/CT was not associated with EFS (T1–2 vs*.* T3–4, log-rank *P* = 0.3). The same observation was made with clinical N score (N0 vs*.* N1–2–3, log-rank *P* = 0.13). Finally, clinical stage determined before PET/CT scanning was not associated with EFS (stage II vs. stage III, log-rank *P* = 0.13).

**Fig. 1:**
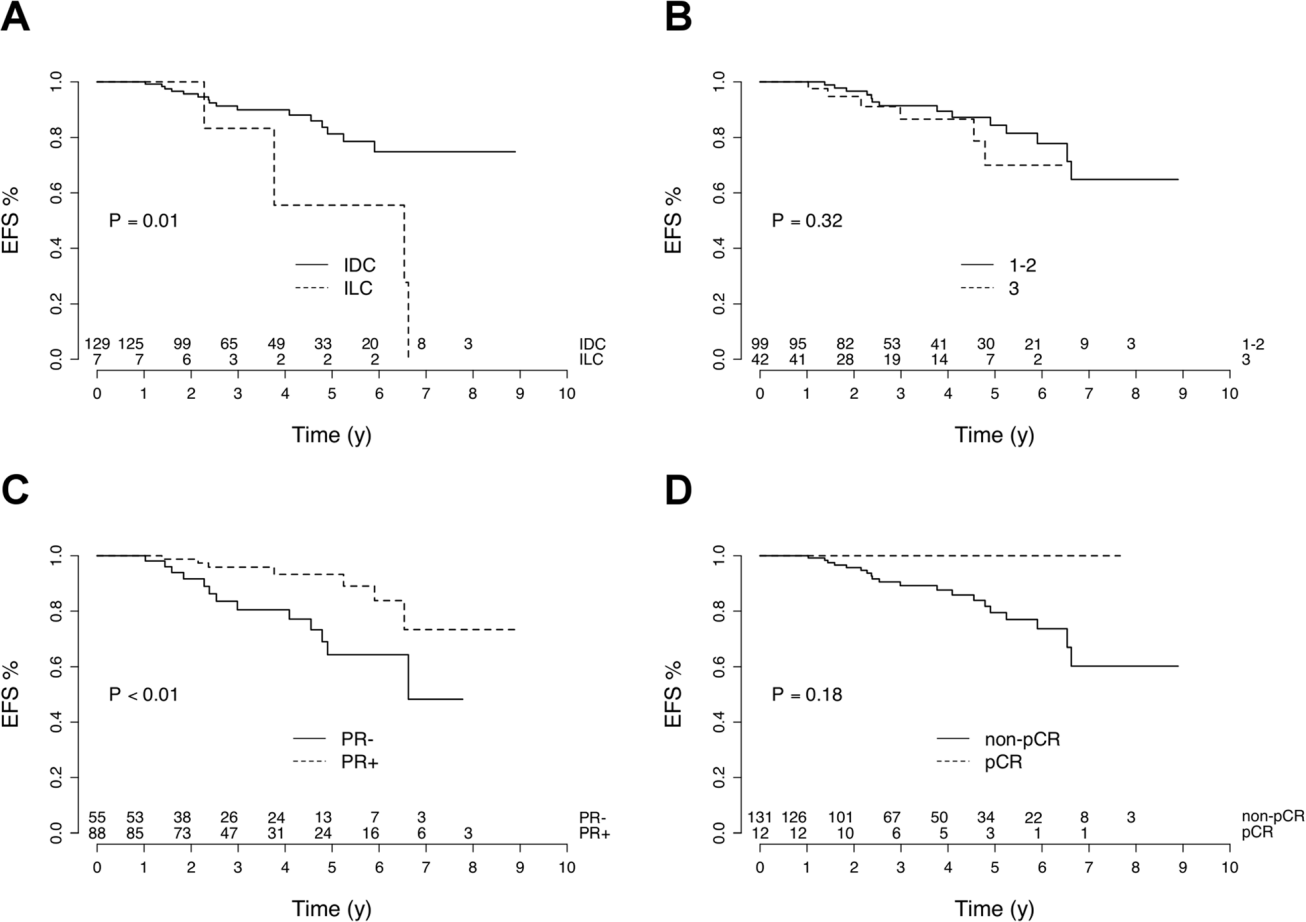
Relation between event-free survival (*EFS*) and histological tumor type (**a**), tumor grade (**b**), progesterone receptor (*PR*) expression (**c**) and pathology findings after neoadjuvant chemotherapy (**d**), in 143 patients with ER+/HER2- breast cancer. *IDC* invasive ductal carcinoma, *ILC* invasive lobular carcinoma, *pCR* pathological complete response

Among baseline tumor characteristics determined at initial biopsy, progesterone receptor expression was associated with EFS, with higher risk of relapse in patients with PR- tumors (HR = 3.25, log-rank *P* < 0.01; Fig. 1). Patients with ILC had shorter EFS in comparison to patients with IDC (HR = 3.74, log-rank *P* = 0.01). With regard to the seven patients with an ILC, none reached pCR while four relapsed. SBR grade (grade 3 vs. grade 1 + 2;) was not related to EFS (HR = 1.64, *P* = 0.32; Fig. 1).

At completion of NAC, only 12 patients (8%) had pCR. No relapse was observed in the 12 women whose tumors reached pCR (0/12 vs*.* 20/131, *P* = 0.36). However pCR was not found to be significantly associated with EFS (log-rank *P* = 0.18; Fig. 1).

### Relation between PET-derived parameters and EFS

**Fig. 2:**
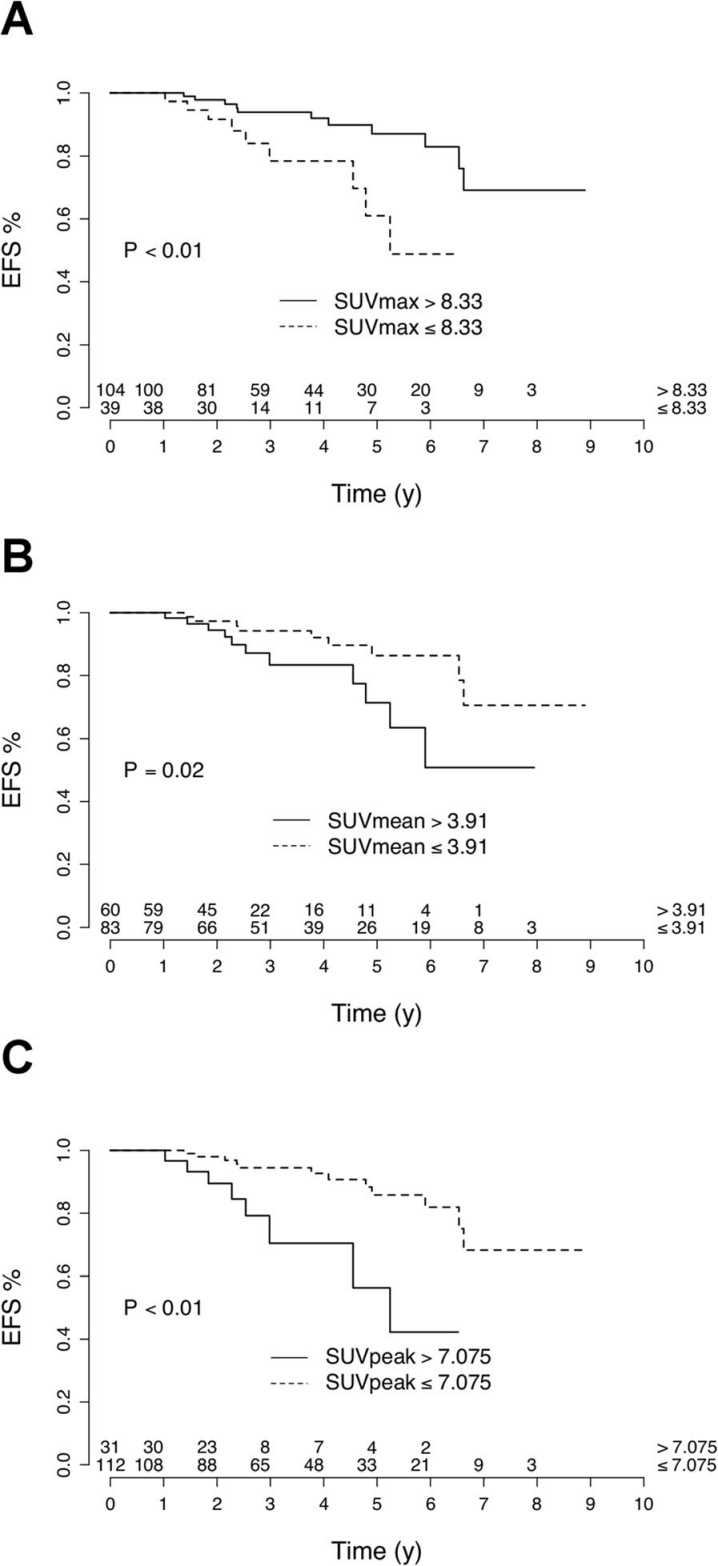
Relation between event-free survival (*EFS*) and baseline tumor standardized uptake value (*SUV*)_max_ (**a**), SUV_mean_ (**b**), SUV_peak_ (**c**) in 143 patients with ER+/HER2- breast cancer

A high SUV_max_ at baseline was associated with shorter EFS (HR = 3.51, log-rank *P <* 0.01; Fig. 2). The Youden index method allowed identifying an optimal cutoff value of 8.3. Nine patients over 39 (23.1%) with baseline tumor SUV_max_ > 8.3 relapsed vs. 11/104 patients (10.6%) with baseline tumor SUV_max_ ≤ 8.3. The 3-year EFS was 78.4% in patients with baseline tumor SUV_max_ > 8.3 (vs. 94.0% in those with SUV_max_ ≤ 8.3). The cutoff value of 10, previously published [5], was also found to be significantly associated with EFS (log-rank *P <* 0.01). High tumor SUV_mean_ and mostly high tumor SUV_peak_ were also associated with shorter EFS (HR = 2.76, *P =* 0.02 and HR = 4.40, *P <* 0.01, respectively, Fig. 2).

**Fig. 3:**
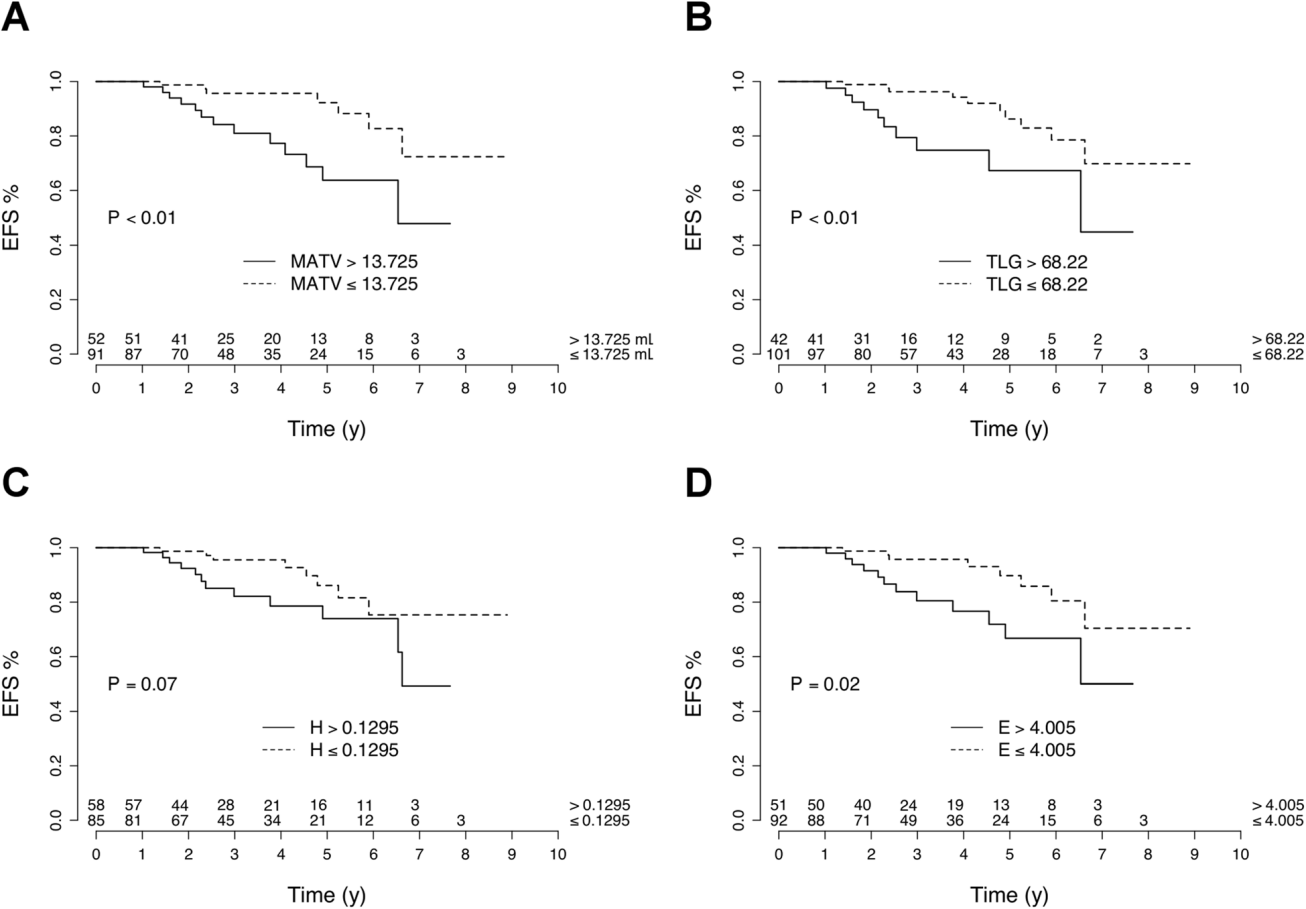
Relation between event-free survival (*EFS*) and baseline tumor metabolically active tumor volume (*MATV*) (**a**), total lesion glycolysis (*TLG*) (**b**), homogeneity (*H*) (**c**), and entropy (*E*) (**d**) in 143 patients with ER+/HER2- breast cancer

Tumor volume parameters were also predictive of EFS, with higher risk of recurrence in the case of larger tumor (HR = 3.47 and *P* < 0.01 for MATV; HR = 3.10 and *P* < 0.01 for TLG; Fig. 3).

Among tumor texture parameters, entropy was predictive of EFS (HR = 2.89, log-rank *P =* 0.02) while homogeneity did not have a significant value (HR = 2.27, log-rank *P =* 0.07) (Fig. 3)*.*

The associations between SUV_max_, SUV_peak_, SUV_mean_, MATV, TLG, and entropy with EFS remain significant after Hochberg’s procedure.

### Comparison of PET parameters and tumor characteristics to predict EFS

A Cox proportional hazard regression model with PET parameters and tumor characteristics was used for univariate analysis to predict EFS (Table 3). The clinical parameters age (*P* = 0.10), grade (*P* = 0.60), tumor classification (*P* = 0.09), lymph node classification (*P* = 0.24) and stage (*P* = 0.05, not significant after multiple testing correction) were not prognostic factors of EFS in univariate analysis and then not included in the multivariate model. The followings parameters were significantly associated with EFS on univariate analysis, after multiple testing corrections (Benjamini-Hochberg procedure): histology (*P* = 0.02), PR expression (*P* = 0.01), SUV_max_ (*P* < 0.01*)*, SUV_mean_ (*P* = 0.01*)*, SUV_peak_ (*P* < 0.01*)*, MATV (*P* < 0.01*)*, TLG (*P* < 0.01*)*, entropy (*P* = 0.02), and homogeneity (*P* = 0.02*)*. All those parameters were subjected to multivariate analysis. Three parameters remained significantly associated with EFS in multivariate analysis (Table 3): MATV (HR = 1.01, *P* < 0.01), progesterone receptor expression (HR = 2.90, *P =* 0.03) and tumor histology (HR = 3.80, *P =* 0.02).

**Table 3:** Uni- and multivariate analysis using metabolic PET parameters and clinicopathologic factors for EFS

Parameters	Univariate analysis	Multivariate analysis	
	*P* value	*P* value	Hazard ratio (95% CI)
Age	0.10	Not included	
Histology	**0.02**	**0.02**	3.80 (1.22–11.80)
Grade	0.60	Not included	
PR expression	**0.01**	**0.03**	2.90 (1.12–7.49)
Tumor classification	0.09	Not included	
Lymph node classification	0.24	Not included	
Stage	0.05^a^	Not included	
SUV_max_	**<0.01**	Not retained in the final model	
SUV_mean_	**0.01**	Not retained in the final model	
SUV_peak_	**<0.01**	Not retained in the final model	
MATV	**<0.01**	**<0.01**	1.01 (1.00–1.02)^b^
TLG	**<0.01**	Not retained in the final model	
Entropy	**0.02**	Not retained in the final model	
Homogeneity	**0.02**	Not retained in the final model	

*P* value corresponds to Cox proportional hazard regression model. Hazard ratios were calculated with their 95% confidence interval (CI). Bold numerals correspond to statistically significant *P* values

*PET* positron emission tomography, *EFS* event-free survival, *PR* progesterone receptor, *SUV* standardized uptake value, *MATV* metabolically active tumor volume, *TLG* total lesion glycolysis, *H* homogeneity, *E* entropy

^a^Not significant after multiple testing correction

^b^HR for MATV was calculated on a continuous variable (if MATV increase from 1 ml, the risk of relapse increase of 1%)

## Discussion

In patients with breast cancer of mixed phenotypes, several teams observed that the change in SUV values early during neoadjuvant treatment could be a good indicator of pathological response and potentially outcome [8, 17]. However, others studies suggested that baseline ^18^FDG uptake, which would avoid performing a second examination, could also be of interest to predict patient outcome, especially in ER+/HER2- BC [4, 5]. Our study was designed to evaluate the predictive value of novel PET parameters based on texture analysis (entropy and homogeneity). It is unclear what the significance of the textural modification under treatment is and how to measure this change. Therefore we decided to focus our study on the predictive value of PET parameters measured at baseline.

ER+/HER2- BC has less intense ^18^FDG uptake than some other phenotypes such as TN carcinoma [2, 18]. Nevertheless, several PET-derived indices measured before treatment were predictive of event-free survival in our cohort of 143 patients with no evidence of distant metastases at initial evaluation. In those patients, the extent of pathological response measured after the neoadjuvant chemotherapy was not predictive of EFS. Complete pathological response to NAC is a rare event in ER+/HER- breast cancer (only 8% of patients in the present series). Although no relapse was observed in the 12 women whose tumors reached pCR (0/12 vs*.* 20/131, *P* = 0.36), pCR was not found to be significantly associated with EFS (log-rang *P* = 0.18; Fig. 1).

Among baseline tumor characteristics, tumor grade was not predictive of EFS. Progesterone receptor negativity was associated with a higher risk of relapse, as in a previously reported series [19]. Histological type was also associated with patient outcome with shorter EFS in the case of invasive lobular carcinoma in comparison to ductal carcinoma (log-rank *P* = 0.01). Loibl et al. observed that patients with ILC had a low chance of obtaining a pCR although it was not well correlated with further outcome [20]. In another study focusing on the ER+/HER2- subgroup, patients with ILC had shorter disease-free survival and overall survival than patients with IDC [21], in congruence with our data.

Patients with high baseline ^18^FDG tumor uptake are at higher risk of early recurrence. The 3-year EFS was 78.4% in patients with baseline tumor SUV_max_ > 8.3 (vs. 94.0% in those with SUV_max_ ≤ 8.3). However, in multivariate analysis, MATV was the solely PET parameter significantly associated with EFS (Table 3). Son et al. observed also that MATV was predictive of patient outcome [10]: in 123 patients with IDC, among four PET parameters (SUV_max_, MATV, TLG, and heterogeneity), only MATV and heterogeneity were predictive of overall survival [10]. However, the heterogeneity factor used was defined as “a derivative of a volume threshold function from 40% to 80% of the SUV_max_” and was reported to be highly correlated with MATV (r = 0.96) [10]. This factor was only a surrogate measurement of volume and it cannot be considered as a measurement of intratumor heterogeneity. Parameters that characterize the tumor texture did not further improve the prediction of EFS in our study.

We determined MATV according to an adaptive threshold method [12]. This approach has demonstrated high accuracy, robustness, and reproducibility [12]. Our results might have been different if we had used a less accurate method such as fixed threshold-based approaches. In a group of 142 women with breast cancer of mixed phenotypes, it was found that SUV_max_ had superior predictive value than volume parameters (namely TLG) derived through manual contouring and fixed threshold [22].

Our study has some limitations. This was a single institution experience. The follow-up period was still limited (median = 44 months) when considering the fact that many recurrences in patients with ER+/HER2- tumors occur between 5 and 10 years after treatment, or even later.

We included patients with large tumors. Our results could not be observed in small tumors, for which partial volume effect has a significant impact in SUV value measurement.

We focused our analysis on ER+/HER2- breast cancers. This subgroup is based on immunohistochemistry tests and defines patients receiving a homogeneous treatment regimen [5, 7, 8]. Others categorizations could have been considered and, in particular, ER+ BC can also be dichotomized into luminal A (ER+/HER2- breast tumor, with low grade and low proliferation) and luminal B (which regroups high proliferative ER+/HER2- breast carcinoma and some ER+/HER2+ tumors). We decided to restrict analysis to ER+/HER2- breast cancer and excluded HER2+ tumors because these patients receive specific targeted treatments (trastzumab) and have specific ^18^FDG-PET response characteristics to NAC [23–26].

Optimal PET parameter cutoff values for predicting EFS were determined at 3 years of follow-up with the Youden index after receiver-operating characteristics curve analysis. Our thresholds are specific of our population of ER+/HER2- BC patients. At baseline, ^18^FDG uptake depends on the breast cancer phenotype [2]. For example, triple-negative breast cancer has higher uptake than luminal breast tumors. Therefore, the thresholds of SUV values should be potentially higher in the case of triple-negative tumors.

We focused our analysis on seven semi-quantitative metabolic PET-derived parameters calculated from static acquisitions. Fully quantitative kinetic analysis may also be helpful for therapeutic response prediction [27] but, kinetic measurements are much more difficult to apply in clinical practice.

Finally, some PET tracers other than ^18^FDG could also be of interest in BC. In particular, 16α-^18^F-fluoro-17β-estradiol (^18^FES) could be efficient in luminal tumors [28].

## Conclusions

In a series of 143 patients, our study confirms that baseline PET parameters measured before neoadjuvant treatment have prognostic values in ER+/HER2- locally advanced breast cancer patients. After multivariate analysis, metabolically active tumor volume remains significant while textural analysis of PET images is not of added value. These findings suggest that ^18^FDG PET image-derived indices, notably volume parameters, may be helpful to plan patient follow-up, as well as to select high-risk patients within trials investigating novel treatment strategies.

Considering histopathological parameters, our study shows that patients with PR-negative or lobular invasive tumor have poorer prognosis than patients with PR-positive or ductal carcinoma, respectively.

## Abbreviations

^18^FDG: ^18^F-fluorodeoxyglucose
BC: breast cancer
CI: confidence interval
E: entropy
EC-D: four cycles of epirubicin + cyclophosphamide followed by four cycles of docetaxel at conventional doses
EFS: event-free survival
ER: estrogen receptor
H: homogeneity
HER2: human epidermal growth factor receptor 2
HR: hazard ratio
IDC: invasive ductal carcinoma
ILC: invasive lobular carcinoma
MATV: metabolically active tumor volume
NAC: neoadjuvant chemotherapy
pCR: pathological complete response
PET/CT: positron emission tomography/computed tomography
PR: progesterone receptor
ROI: region of interest
SBR: Scarff-Bloom-Richardson
SUV: standardized uptake value
TLG: total lesion glycolysis
TN: triple-negative
